# Spatial Hyperschematia without Spatial Neglect after Insulo-Thalamic Disconnection

**DOI:** 10.1371/journal.pone.0079938

**Published:** 2013-11-26

**Authors:** Arnaud Saj, Juliane C. Wilcke, Markus Gschwind, Héloïse Emond, Frédéric Assal

**Affiliations:** 1 Department of Neurology, University Hospital of Geneva and Faculty of Medicine, University of Geneva, Geneva, Switzerland; 2 Laboratory for Neurology and Imaging of Cognition, Department of Fundamental Neurosciences, University of Geneva, Geneva, Switzerland; 3 Department of Clinical Neurosciences, Neurology Service, Vaudois University Center Hospital, University of Lausanne, Lausanne, Switzerland; University of Bologna, Italy

## Abstract

Different spatial representations are not stored as a single multipurpose map in the brain. Right brain-damaged patients can show a distortion, a compression of peripersonal and extrapersonal space. Here we report the case of a patient with a right insulo-thalamic disconnection without spatial neglect. The patient, compared with 10 healthy control subjects, showed a constant and reliable increase of her peripersonal and extrapersonal egocentric space representations - that we named spatial hyperschematia - yet left her allocentric space representations intact. This striking dissociation shows that our interactions with the surrounding world are represented and processed modularly in the human brain, depending on their frame of reference.

## Introduction

Euclidian space extends continuously to infinity, yet appears to be heterogeneously represented in the brain. Embodied space can be divided into at least three regions: personal space, peripersonal space and extrapersonal space [1]–[3]. Personal space is the physical space in which one can feel a sensation, either from a contact or not, or where an activity takes place on the body, such as scratching an itch. Peripersonal space is the working space within arm's reach. Extrapersonal space is the space beyond peripersonal space.

These three nested spaces are coded by specific cognitive networks in different neuroanatomical locations in the brain. Support for these highly segregated networks derives from both animal neurophysiology and human neuropsychology. Single-cell studies in the monkey have shown that neurons in the medial and lateral intraparietal area respond to stimuli in peripersonal and extrapersonal space, respectively [4]. Dissociations between personal, peripersonal and extrapersonal space have also been described in patients with lesions of the right hemisphere, in particular with spatial neglect [3]. Neglect patients typically fail to explore the left side of space, a symptom most frequently encountered after right brain damage (for a review see [5]). A related distinction between near and far space concerns actions and action-properties of stimuli that are specifically related to near space, with subdivisions between body, grasping and locomotion spaces [6].

The hypothesis that the brain develops segregated circuits dedicated to these different spaces seems reasonable if one considers, for example, that an action being spatially oriented towards a goal implies that it is associated with spatial coordinates in a particular framework within the motor system [7]. These regions can be regarded as landmarks that define the different directions of space and as frameworks used to perform movements or perceive space. Each framework consists of a reference origin and unit vectors that define different spatial directions. Three distinct frameworks can be defined according to their reference: 1) a framework that refers to the gravity vector, 2) an allocentric framework that refers to objects, and 3) an egocentric framework that refers to the own body.

Based on the animal and human data mentioned above, one could hypothesize that dissociations between these spatial networks could be shown in brain-damaged patients without spatial neglect, based on the comparison of their space perception per se. Here we report the case of a patient with intact allocentric space representation, but an abnormal extension of represented peripersonal and extrapersonal space in relation to the body. We first designed three behavioral experiments to accurately measure the phenomenon in our patient and compare it to matched controls. Then, we performed diffusion-tensor imaging (DTI) in order to better understand the neural underpinning of this new phenomenon.

## Materials and Methods

### Patient and healthy controls

**Figure 1:**
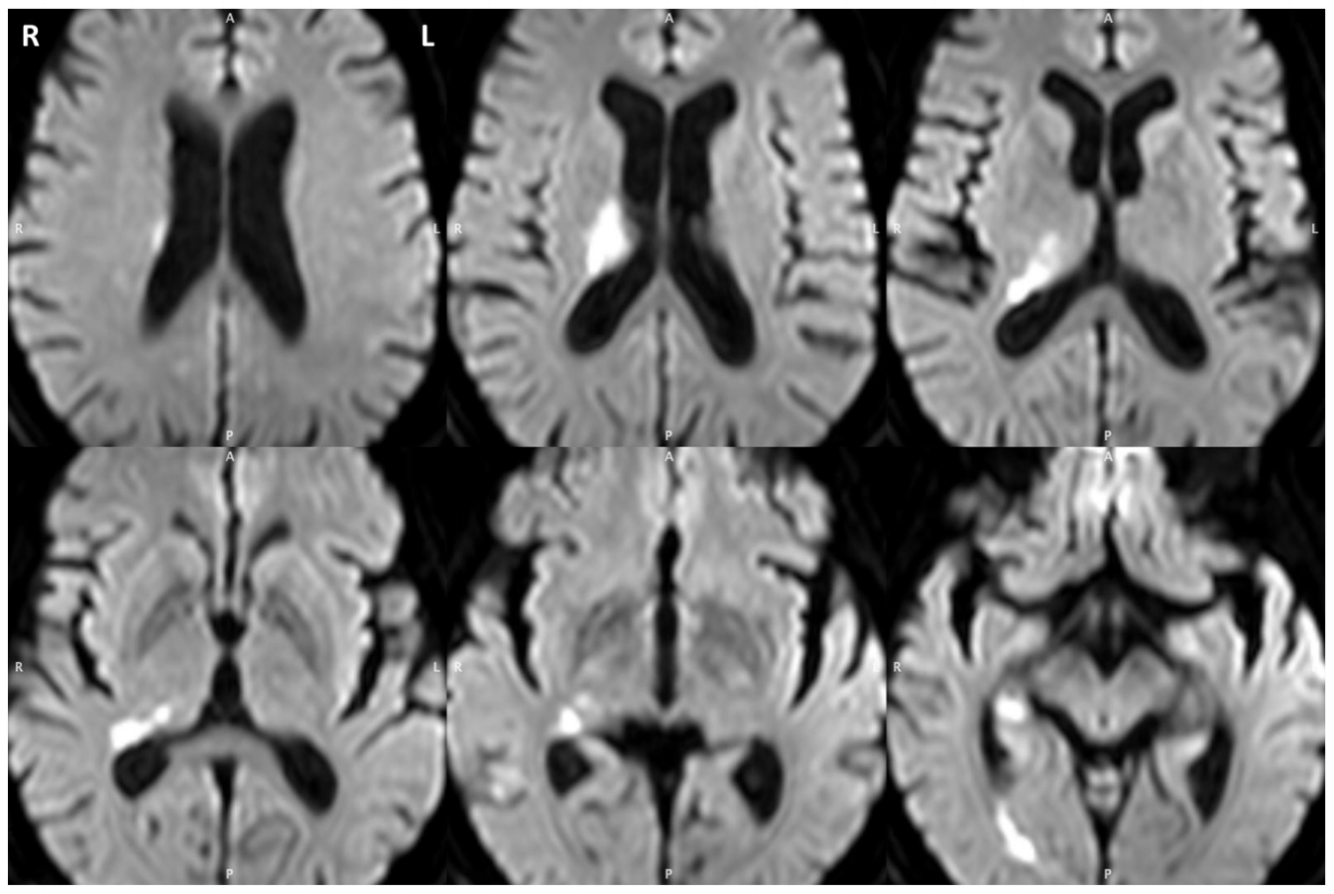
The patient's acute ischemic lesion. The figure shown on axial slices of the diffusion weighted image, involves the right ventrolateral posterior thalamus, the lateral edge of the pulvinar and the adjacent white matter down to the right hippocampus.

T.R., a 83-year-old right-handed woman, was recruited from the Neurology Service at the Department of Clinical Neuroscience in the Geneva University Hospital. She was admitted for left-hand hemiplegia that revealed acute ischemic lesions in the right thalamic region on MRI (see Fig. 1). No identifiable cause was found apart from high blood pressure, and she was discharged with left superior quadranopsia. Immediately after being discharged, she complained about an abnormal feeling of distance from the real environment. Neuropsychological functions were examined using a routine battery of standardized clinical tests, including the Mini-Mental State Examination, in order to exclude dementia and other major cognitive disorders that would affect task performance and collaboration. The results of all neuropsychological tests were normal (see Table 1) and her instrumental activities of daily living entirely intact. Only the daisy drawing test (e.g. flower) showed superficial hyperschematia, with 8 petals on the left compared to 5 on the right. All evaluations were done as usual (i.e. only in the peripersonal space).

**Table 1:** T.R.'s neuropsychological test scores.

			T.R.'s score	Norm			T.R.'s score	Norm
**Language**					**Executive function**			
Boston naming test (/34)			34	>32	Stroop test (min)			
					Time	Color	15″53	>pec 75
Fluency test (2min)	semantic		54	27.14±8.53		Word	18″72	>pec 75
	lexical		38	19.28±7.05		Interference	30″63	>pec 99
					Error	Color	0	>pec 75
**Apraxia**						Word	0	>pec 75
	ideomotor		5	>4		Interference	0	>pec 75
	ideational		6	>5				
	constructional		6	>5	Trail making test			
					Time	version A	104	63.46 (29.22)
**Agnosia**						version B	201	140.54 (75.38)
	categorical		10	>7	Error	version A	0	
	functional		10	>7		version B	0	
								
**Memory**					**Neglect**			
Span	verbal		6	>6	Bell cancellation test			
	spatial		7	>6		Total omissions	6	<6
						Left-Right omissions	−2	<2
16 words		free	cued					
	Recall 1	14	16	pec. 99	Line bisection			
	Recall 2	16	16	pec. 99		% of deviation	2.5%	<11%
	Recall 3	15	16	pec. 99				
	Delayed Recall	13	16	pec. 99	Bisiach test		0	0
	Recognition	16		pec. 99				

Language: Boston naming test [45], Fluency test [46]; Apraxia: sub-test CAMCOG [47]; Agnosia: Protocole Montréal-Toulouse d'Evaluation des Gnosies Visuelles [48]; Memory: Span (sub-test CAMCOG), 16 words (Grober and Buschke test, [49]); Executive function: Luria test [50], Stroop [51], Trail Making Test [52]; Neglect [53]. Percentil: per.

Later on, T.R. constantly reported that very unusual feeling of remoteness of her environment and/or would claim to perceive everything around her as far away. The phenomenon had actually appeared abruptly after the cerebrovascular event, remained stable and chronic when standing or sitting, but strikingly diminished in the supine position.

Healthy participants were recruited through advertisements in the University of Geneva and included 5 female and 5 male volunteers (mean age  = 79.4 years, SD = 7.7 years, range  = 68–93 years), all right-handed and without any history of previous neurological or psychiatric disease. They had normal or corrected-to-normal visual acuity. The study protocol was approved by the local ethics committee (Ethic Central Commission of the University Hospital of Geneva), following the ethical principles expressed in the Declaration of Helsinki. The patient as well as all volunteers were compos mentis and signed written consent forms before taking part in the experimental testing sessions. The examiners explained the purpose of the research to the patient as well as all volunteers and responded to questions and concerns.

### Behavioral tasks

#### Egocentric reachable task

This task served to estimate the perception of action space, operationalized by the farthest target position perceived as reachable with an extended arm. Visual targets were red points 7 mm in diameter, which were presented by the experimenter directly in front of the subject in the frontal plane at any of 25 possible target positions spaced 1 cm apart. The maximum attainable distance was initially estimated by asking sitting participants to extend their arm as far as possible in front of them without moving their upper body (on average 60 cm). Targets were randomly presented twice at each position to make up a total of 50 trials.

#### Egocentric distance task

Standing subjects had to estimate the horizontal distance between them and the experimenter in front of them. The experimenter stood at distances of 20, 50, 90, 120, 150, 190 or 210 cm in random order.

#### Allocentric distance task

The task required subjects in a sitting position to estimate the horizontal distance between two objects (wooden cubes), both of which were placed 90 cm in front of the participant. The distance varied randomly between 5, 10, 15, 20, 30, 40, 50 and 60 cm, and the task was performed in the frontal and lateral plane.

Participants performed five trials per condition. Trials were separated by about 20 seconds, and the tasks by about 2 minutes.

### Procedure

In the first experiment (egocentric reachable task), participants were installed in front of a line of red points. Then the experimenter pointed to one of them at the time, and participants were asked to say whether they thought they could reach it. The actual border (initial point) between reachable and unreachable points was 60 cm, corresponding to the mean distance of the extended arm. In the second experiment (egocentric distance task), participants were asked to estimate the distance between their own body and the experimenter positioned in front of them beyond arm's reach. In the last experiment (allocentric distance task), participants were asked to estimate the distance between two objects placed in extrapersonal space (in the frontal and the lateral plane). In all tasks, participants gave their distance estimates verbally.

### Statistical analysis

We used parametric tests ([8]; the Student t test or Bayesian hypothesis test [http://www.abdn.ac.uk/~psy086/dept/psychom.htm]) to compare the continuous dependent variables (estimated distances in cm) between patient and control subjects.

### DTI data acquisition and analysis

Images of the patient's brain at the acute stage were acquired using a 1.5 T whole-body MRI scanner (Achieva, Philips, Best, the Netherlands) at the University Hospital Geneva, Switzerland, with a 6-channel head coil. We obtained diffusion-weighted images with an echo-planar spin-echo pulse sequence (TR = 3263 ms; TE = 68 ms; flip angle  = 90°; acquisition matrix  = 112×88; 24 slices with a gap of 1 mm; 0.9×0.9×5.0 mm^3^; 3 averages; PAT factor 2). After 19 months we acquired diffusion-tensor images of the patient's brain using a 3 T whole-body MRI scanner (Trio Tim, Siemens, Erlangen, Germany) with a 12-channel head coil at the same hospital, this time employing an echo-planar pulse sequence (TR = 8300 ms; TE = 83 ms; flip angle  = 90°; acquisition matrix  = 128×128; 64 slices; 2×2×2 mm^3^; PAT factor 2). We performed diffusion weighting along 63 independent directions (b = 1000 s/mm^2^) and additionally acquired 10 reference images (b = 0 s/mm^2^).

All DICOM files were converted to NIfTI format using MRIConvert (http://lcni.uoregon.edu/~jolinda/MRIConvert/). We first corrected the chronic diffusion-tensor images for eddy currents and head motion using FDT (part of FSL 4.1; http://fsl.fmrib.ox.ac.uk/fsl/fslwiki/FSL) and then performed data reconstruction and fibre tracking using the Diffusion Toolkit (DTK; http://trackvis.org/dtk/). We next isolated the acute thalamic lesion by thresholding the diffusion-weighted image volume and manually removing unconnected above-threshold voxels. We then used FLIRT (also part of FSL 4.1) to coregister the acute diffusion-weighted image to the chronic diffusion-weighted image ouput by DTK and applied the resulting transformation matrix to the lesion image. After thresholding the latter to restore the size of the lesion's volume after coregistration, we used TrackVis (http://trackvis.org/) to visualise the fiber tracts passing through the acute right thalamic lesion.

## Results

### Behavioral results

In summary, we found a measurable extension of the perception of certain types of space in a brain-damaged patient compared to normal controls. In line with the hypothesized division of spatial representations, the patient differed from the control group in both egocentric tasks but not in the allocentric task (neither in the frontal nor lateral plane).

**Figure 2:**
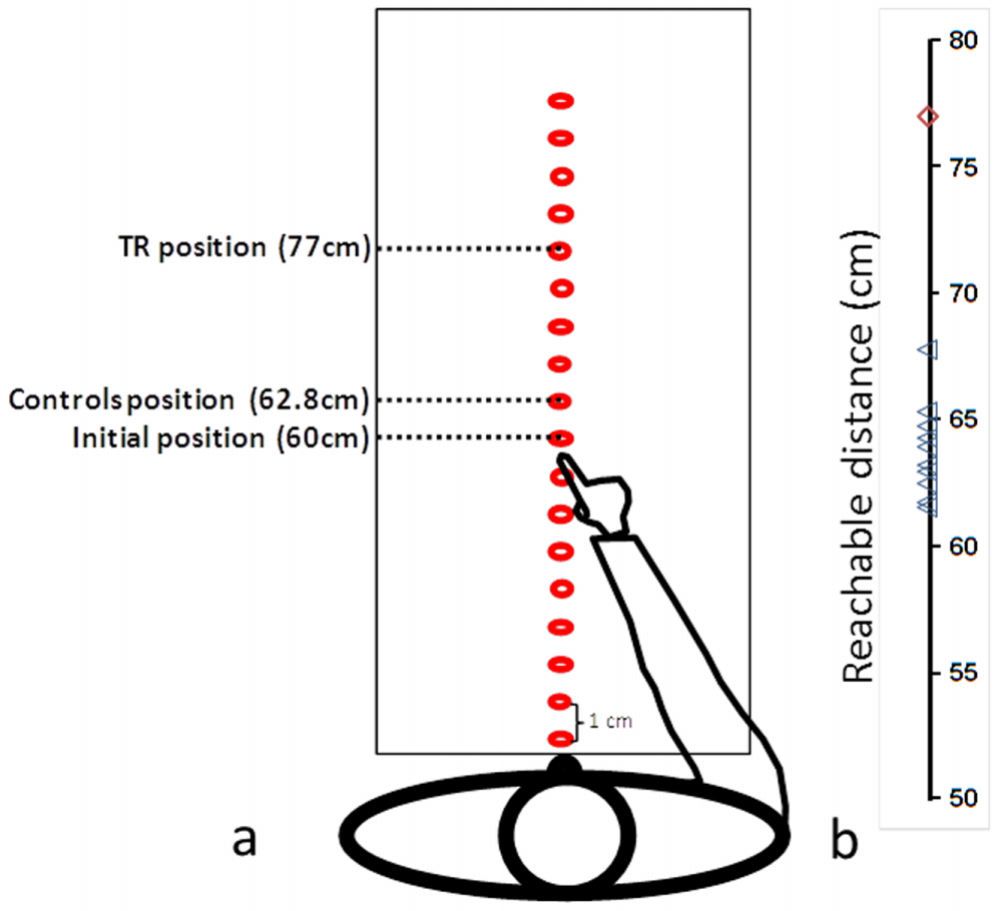
Egocentric reachable task. (a) schematic setup and illustration of the mean distance estimates of reachable stimuli; (b) estimates of T.R. (diamond) and control group (triangles) of the border of their peripersonal egocentric space.

For the egocentric reachable task (figure 2), T.R.'s distance estimates (mean  = 43.0±1.8 cm) were significantly shorter than those of the control group (one-tailed probability  = 0.002). The estimates of the latter were similar to previously reported data [9], whereas the patient's perceived reachable point was unequivocally shifted closer to her body, in line with peripersonal space being perceived as being further away.

**Figure 3:**
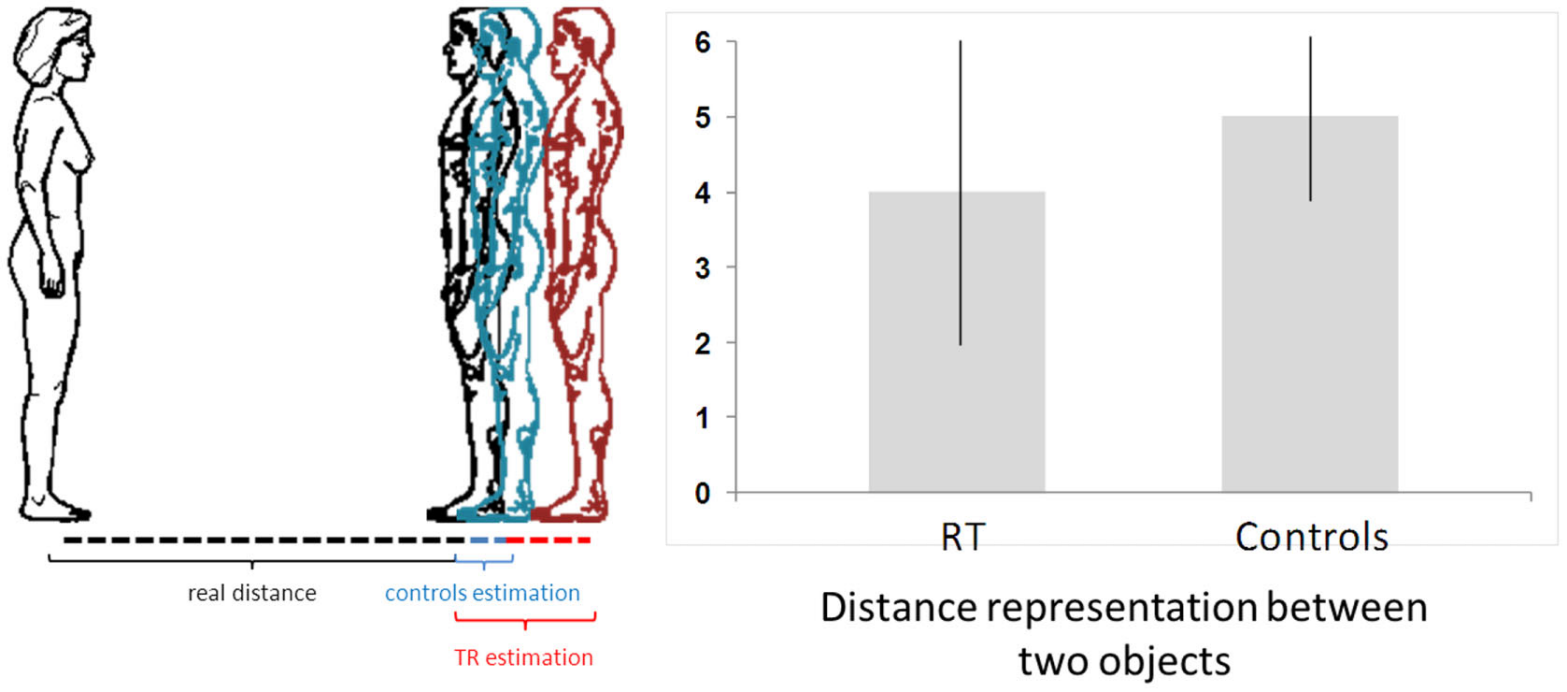
Egocentric distance task. (a) schematic setup and illustration of the mean distance estimates between participants and experimenter; (b) mean estimates of T.R. and control group of a distance in their extrapersonal egocentric space.

For the egocentric distance task (figure 3), T.R.'s estimates also showed a significant difference compared to those of the control group (one-tailed probability  = 0.04). The latter overestimated the real distance by 5.2±8.2 cm, whereas the patient did so by 21.4±8.6 cm. T.R. thus showed an extension of more than 20 cm of both her peripersonal and extrapersonal space.

In the allocentric distance task, the patient showed no significant difference compared to the control group in the two dimensions. In the lateral plane, deviations from the actual distance between two objects were 4.1±1.0 cm and 5.8±2.4 cm (one-tailed probability  = 0.25), respectively. In the frontal plane, they were 2.3±0.5cm and 3.2±0.9 (one-tailed probability  = 0.18). T.R. had therefore no difficulty in estimating the distance between two objects placed in her extrapersonal space.

### DTI results

**Figure 4:**
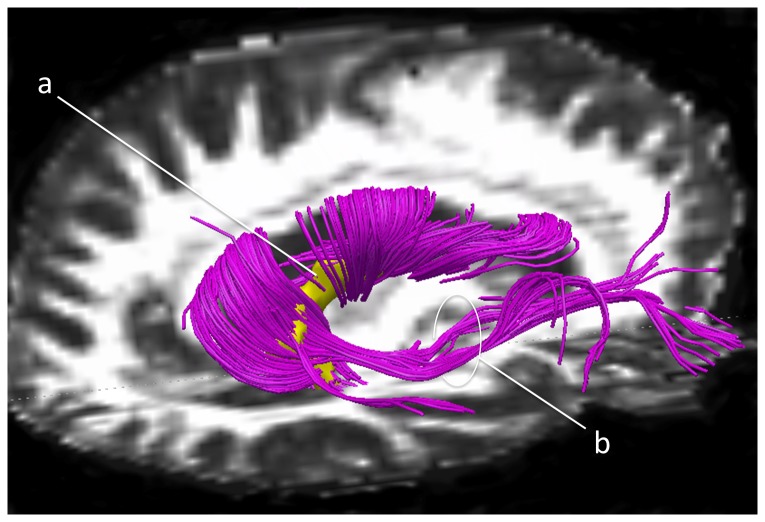
The fiber tracts affected by the patient's lesion. The patient's acute lesion (a, yellow) and affected fibre tracts (purple) in the chronic brain, superimposed on the patient's fractional anisotropy image. The tracts include at the posterolateral edge of the pulvinar the inferior fronto-occipital fasciculus (b), which projects to the external capsule and the insula.

The fiber tracts affected by the patient's lesion (figure 4a) project from the ventrolateral posterior nucleus of the right thalamus to the corpus callosum and frontal areas, and involve, at the posterolateral edge of the pulvinar, the inferior fronto-occipital fasciculus (figure 4b), which originates from the lingula and inferior parietal regions and projects to the external capsule and the insula.

## Discussion

Our data show for the first time an extension of peripersonal and extrapersonal egocentric space in a patient with vascular lesions in the right hemisphere, specifically disconnecting the right posterior thalamus from the insula. Her perception of peripersonal distances was increased by over 17 cm compared to the corresponding physical distances, and her perception of extrapersonal distances by more than 20 cm. However, these extensions of space were present only when she was estimating a distance from her body, but not when estimating the distance between two objects. These results perfectly fit with the patient's complaint of remoteness of the environment.

Such a distortion of space has, to our knowledge, never been reported in the literature. Other authors [10]–[11] have carried out direct tests for perceptual distortions in neglect patients by asking them to make matching judgments of pairs of horizontal and vertical rectangles or nonsense shapes. Their results showed significant and substantial underestimations of the area of stimuli presented on the left side of egocentric space, but no such misperceptions of stimuli presented along the vertical meridian, and were interpreted in terms of attentional disengagement from an image junction and diminished strength of the representation of objects within the neglected side [12]. Nevertheless, these studies concern the perception of distance between two objects without any reference to the perceiver's body. Studies on spatial perception with reference to the perceiver's body found that patients with neglect syndrome showed a shift towards the ipsilesional side [3] and a compression of the left contralesional space [13].

Specific areas of extrapersonal and personal space have been identified in the right hemisphere at the level of the central sulcus, in the inferior frontal gyrus and mainly in the insular/opercular regions of the temporo-peri-sylvian cortex [14]. The latter may correspond to the cortical vestibular region [15], which is thought to contribute to the egocentric representation of space by integrating different sensory inputs [16]. Recent findings in patients with brain lesions indicate that the right insular cortex may play a crucial role in the genesis of our self-awareness of limb movement and our sense of limb ownership [17]–[18]. The authors of those studies speculated that the right insula might be a core structure of a network involved in human body scheme representation and self-awareness of own body parts.

Another primary role of the insula is the perception of space verticality [19]–[20], or the posture [21] and higher-order spatial integration of vestibular information [22]. Lesions in other anatomical regions than the insula may induce tilts of the perception of verticality [19], [21], [23], [24], especially in the temporal cortex, the parietal cortex and the thalamus [21], [23], [24]. The relationship between lesion extension and abnormalities in verticality perception indicates that verticality representation depends more on the competencies of neural circuits than on the specificity of a given brain structure. Relevant neural circuits might include the thalamo-parietal projections for the perception of egocentric space and the thalamo-insular projections for the perception of verticality [25].

The existence of such a subjective visual vertical perceived as tilted towards body tilt (known as the “Aubert effect”) requires the integrity of the posterolateral thalamus, which plays a functional role in the processing of both vestibular [26] and somaesthetic graviception [21]. This role is supported by the existence, in humans, of projections from the anterior part of the pulvinar and from its ventroposterior lateral nuclei to the parieto-insular, temporal and parietal cortices [27], [28]. These multisensory integrations have been suggested to be processed in the parieto-insular vestibular cortex [29], which is located near the posterior end of the insular cortex in monkeys. Neuroimaging studies suggest that the posterior insula and surrounding areas form the core vestibular cortex in the human brain [30], [31]. Lesions in the peripheral vestibular system such as the vestibular nuclei and the ventral postero-lateral thalamic nucleus, which receives vestibular inputs, can all alter parieto-insular vestibular cortex activity [32]. The right parieto-insular vestibular cortex could be responsible for multimodal interactions/integrations concerning one's own body [33].

A recent study [34] found this structure was strongly implicated in the verticality and egocentric perception using voxel-based lesion-symptom mapping among 44 right brain-damaged patients (with or without spatial neglect). Indeed, the results reinforce the implication of parieto-insular regions in the body and vertical perceptions, as well as in postural representation. In sum, our behavioral and neuroanatomical data showing an insulo-thalamic disconnection confirm the existing literature on the role of both the insula and the thalamus in the perception of body and space, either verticality and egocentric spatial frameworks. Such a dissociation between disrupted egocentric versus preserved allocentric representations was already described in a patient with a right thalamic lesion [35]. Egocentric and allocentric space representations are therefore underpinned by cortico-subcortical networks [36].

In conclusion, such a unique enlargement of the egocentric space in T.R. - a phenomenon that has been described here with caution for the first time in the literature - could be called a “hyperschematia of space.” So far the hyperschematia phenomenon has been described as an enlargement of a portion of an object [37], drawing [38], [39] or body without disorders of size perception (object and drawing) [40]. The concept of hyperschematia was originally proposed by the French otolaryngologist Pierre Bonnier in 1905, based on his clinical observations of patients with vestibular disorders (see [41], [42]). These patients reported their body parts as being absent, smaller, bigger or misallocated with respect to their actual positions. Bonnier ascribed these deficits to specific disorders of the topographic schema of the body. He classified the patients as cases of *hyper*- or *hyposchematia*, that is patients with the illusory over- or underestimation of the size of the whole body (or of parts of it). The phenomenon has recently been observed in right-brain-damaged patients, both with spatial neglect [37], [38] and without neglect [39].

The parallel between this phenomenon and the abnormal space representations in our patient also concerns its cortical anatomy. As just mentioned, hyperschematia was first observed in patients with disorders of vestibular origin. Our patient presents with a disconnection between the right posterior thalamus and the right insula in regions that are strongly related to vestibular cortex in monkeys [29], [43]. Studies in the human brain show that this vestibular region closely corresponds to the parieto-insular cortex [44], the neurons of which respond to multimodal stimulations (vestibular, optokinetic and somatosensory) characterizing this region as multisensory. A recent case study [40] seems to have confirmed the role of vestibular inputs in hyperschematia (facial macrosomatognosia). Our data thus indicate that one's interactions with the surrounding world are likely represented and processed in separate modular networks dedicated to egocentric and allocentric spatial representations in the brain and open up new questions about the nature of space representation in humans.
